# The etiologies of post-stroke depression: Different between lacunar stroke and non-lacunar stroke

**DOI:** 10.1016/j.clinsp.2022.100095

**Published:** 2022-08-23

**Authors:** Ke-Wu Wang, Yang-Miao Xu, Chao-Bin Lou, Jing Huang, Chao Feng

**Affiliations:** a The Fourth Affiliated Hospital of Zhejiang University School of Medicine, Yiwu, China; b Shanghai Xuhui Central Hospital, Shanghai, China

**Keywords:** Post-stroke depression, Lesion location, Lacunar stroke, Non-lacunar stroke, PSD, Post-stroke depression, WMH, White matter hyperintensities, SBI, Silent brain infarctions, GDS, Geriatric Depression Scale, LSNS, Lubben Social Network Scale, mRS, Modified Rankin Scale, NIHSS, National Institutes of Health Stroke Scale, DSM, Diagnostic and Statistical manual of mental disorders

## Abstract

**Objectives:**

Depression is common after both lacunar stroke and non-lacunar stroke and might be associated with lesion locations as proven by some studies. This study aimed to identify whether lesion location was critical for depression after both lacunar and non-lacunar strokes.

**Methods:**

A cohort of ischemic stroke patients was assigned to either a lacunar stroke group or a non-lacunar stroke group after a brain MRI scan. Neurological deficits and treatment response was evaluated during hospitalization. The occurrence of depression was evaluated 3 months later. Logistic regressions were used to identify the independent risk factors for depression after lacunar and non-lacunar stroke respectively.

**Results:**

83 of 246 patients with lacunar stroke and 71 of 185 patients with non-lacunar stroke developed depression. Infarctions in the frontal cortex, severe neurological deficits, and a high degree of handicap were identified as the independent risk factors for depression after non-lacunar stroke, while lesion location was not associated with depression after lacunar stroke.

**Conclusion:**

The main determinants for depression after lacunar and non-lacunar stroke were different. Lesion location was critical only for depression after non-lacunar stroke.

## Highlights

• The main determinants for depression after lacunar and non-lacunar stroke were different.
• Infarctions in the frontal cortex were significantly associated with post-stroke depression.
• For patients of lacunar stroke, the location of the infarction was not associated with the presence of post-stroke depression.

## Introduction

Post-Stroke Depression (PSD) is a common consequence after a stroke, with its prevalence of more than 30% as reported.^1^,^2^ PSD has been proven to be associated with poor response to rehabilitation, poor quality of life, and high mortality,^3^, ^4^, ^5^ with the pathogenesis not clearly elucidated. Among the various factors related to PSD, lesion laterality and locations, accumulation of silent lesions, stroke severity and psychosocial factors were most frequently mentioned.^6^, ^7^, ^8^ However, discrepancy about the specific etiology of PSD especially on the role of lesion laterality and location is prominent among different studies. For example, Machale, et al. and Castellanos-Pinedo, et al. both reported that infarctions involving the right cerebral hemisphere were more significantly associated with PSD,^9^,^10^ while some other researchers such as Terroni, et al. and Hama S, et al. found that lesions in the left prefrontal cortex were related to PSD.^11^,^12^ Until now, there is no conclusion about the association between PSD and lesion location.^13^,^14^ The different methodologies might be one reason for the discrepancy. Meanwhile, the authors noticed that few studies made a detailed analysis of the etiologies of depression after different subtypes of stroke, such as lacunar and non-lacunar stroke, which were quite different in lesion location, lesion size,^15^ neurological dysfunction, and functional outcome,^16^ yet were similar in the prevalence of PSD.^17^,^18^ It's possible that the significance of lesion location and the specific lesion location related to PSD might be different between the two subtypes of stroke. In order to test this hypothesis, the authors investigated a cohort of stroke patients and tried to make a detailed analysis of the etiologies of depression after lacunar and non-lacunar stroke respectively.

## Materials and methods

### Subjects

From May 2018 to July 2020, 544 patients with first-ever acute ischemic stroke who attended to the Shanghai Xuhui Central Hospital were consecutively enrolled into the study cohort if the patient met all the criteria: 1) Having the will and ability to give consent to this study; 2) Age more than 18 years old; 3) Being able to undergo MR scan and other clinical evaluation. Patients with the following conditions were excluded: 1) Previous history of ischemic or hemorrhagic stroke; 2) Brain tumor, Parkinson's disease, or other central nervous system diseases; 3) History of depression, anxiety, or drug dependence; 4) Moderate or severe cognitive dysfunction, with a Mini-Mental State Examination (MMSE) score lower than 18; 5) Severe communication problems including severe aphasia or dysarthria; 6) Undergoing thrombolytic therapy or endovascular treatment. Among the 544 patients enrolled, 63 patients were lost to follow-up because of death, movement, or other unknown reasons, and 50 patients had a recurrent stroke or developed severe complications including poor-controlled infection, cardiac arrest, heart failure, and renal failure within the three months after the index stroke, thus were not analyzed in this study. The sample size was calculated to be 530 based on a 30% prevalence of PSD, 95% Confidence Interval, a 2.5% estimation error, and a 20% percentage of loss in follow-up (calculator on medsci.cn). This study was proved by the Ethics Committee of the Shanghai Xuhui Central Hospital, with written consent form participants or their family members.

### Demographic and clinical data

The following information was collected during hospitalization: sex, age, education years, the prevalence of hypertension and diabetes, MMSE scores. Neurological deficits were evaluated at admission and 7^th^ day of hospitalization according to the National Institutes of Health Stroke Scale (NIHSS). An NIHSS score on the 7^th^ day not lower than that at admission was defined as an unfavorable treatment effect. Before discharge, patients were given individualized rehabilitation treatment plans by a rehabilitation therapist.

### Radiological examination

Subjects were scanned mostly by a 1.5 T scanner (Philips, Netherlands), and partly by a 3.0 T scanner (Siemens, Germany). The MRI protocol consisted of a T1-weighted image (Repetition Time/Echo Time ‒ TR/TE = 101/1.92 for 1.5 T, 2000/9 for 3.0 T scanner), Fluid Attenuated Inversion Recovery images (FLAIR) (TR/TE = 6000/110 for 1.5 T, 8500/94 for 3.0 T scanner), and Diffusion-Weighted Images (DWI) (TR/TE = 3393/86 for 1.5 T, 6000/94 for 3.0 T scanner) in the axial plane, as well as a T2-weighted image (TR/TE = 1940/120 for 1.5 T, 4540/96 for 3.0 T scanner) in the sagittal plane with 16 layers.

**Fig. 1:**
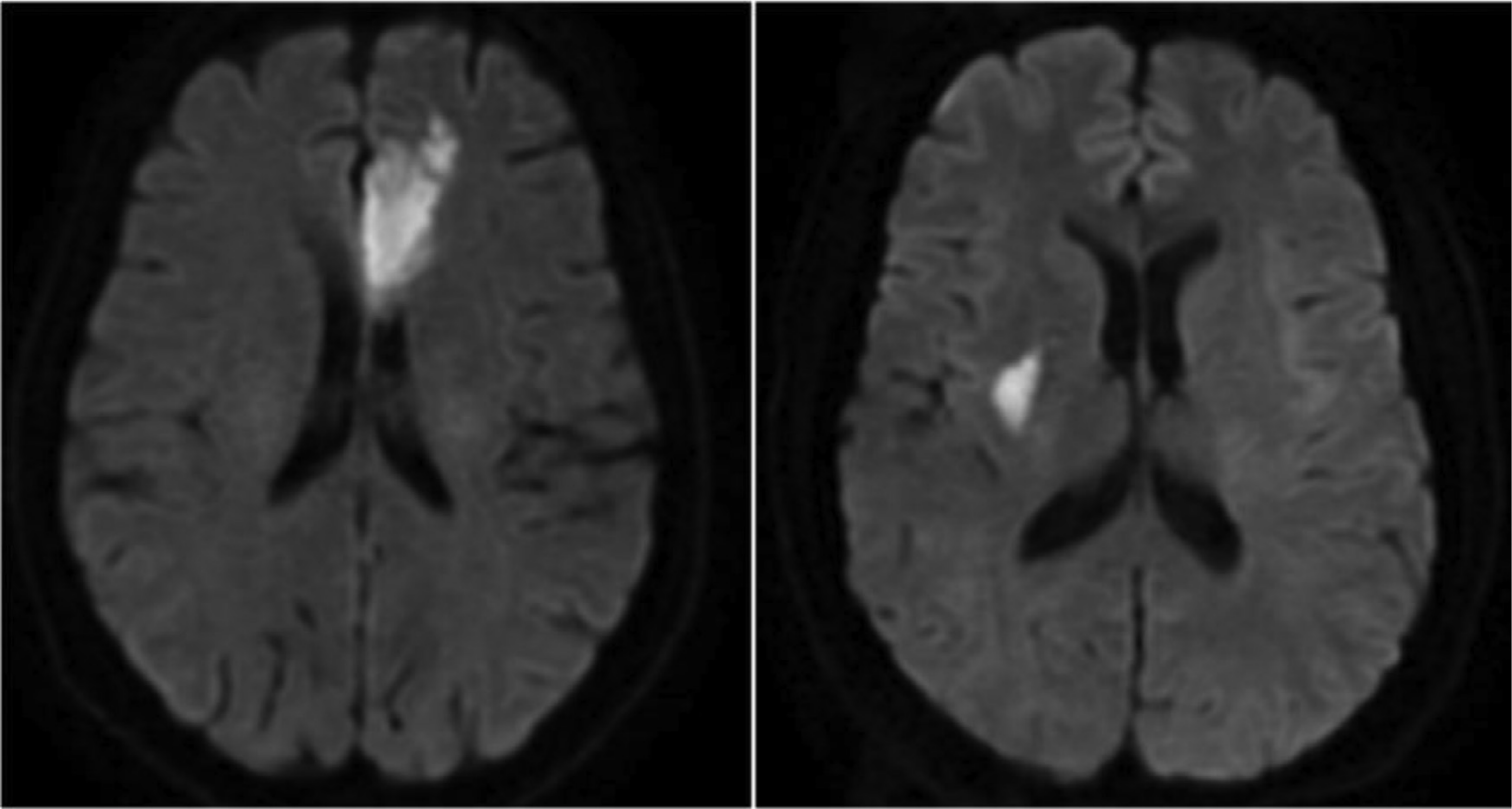
The acute infarctions of two patients of PSD on DWI. Left: A 63-year-old female, she was admitted because of right limb weakness and aphagia with NIHSS score 3. The brain MRI showed acute infarction in left frontal lobe. After hospitalization for 7 days, she was discharged with a NIHSS score 2. Three months later she had no obvious functional impairment but had a GDS score 9 and was identified to have PSD. Right: A 54-year-old male, he was admitted because of weakness and numbness of left limb with NIHSS score 3. The brain MRI showed acute lacunar infarction in right basal ganglia. During hospitalization he didn't react well to the treatment and had neurological deterioration with NIHSS score 5 at the 7^th^ day of hospitalization. Three months later he had a GDS score 10 and was identified to have PSD.

All images were assessed by two radiologists blind to the clinical information. The discrepancy was resolved by a visual consensus. The diagnosis of ischemic stroke was based on the acute neurological symptoms and the visible infarcts on MRI with hyperintense on DWI (Fig. 1). Lacunar stroke was defined as single or multiple acute ischemic infarcts in the perforating-artery territories or subcortical regions, with the longest diameter less than 20 mm on DWI,^19^,^20^ otherwise the patients were deemed to have a non-lacunar stroke. According to the specific diagnosis, patients were assigned to either lacunar stroke group or the non-lacunar stroke group.

The presence of silent lesions including White Matter Hyperintensities (WMH) and Silent Brain Infarctions (SBI) were also evaluated. WMH was defined as focal or confluent hyperintensities in the deep or periventricular area on FLAIR images.^21^,^22^ Periventricular WMH (PWMH) and Deep WMH (DWMH) were respectively graded as 0 to 3 according to Fazekas’ scale.^23^ Infarcts with a > 3 mm-diameter, hypointense on T1-weighted images, and hyperintense on T2-weighted images, without a corresponding history of stroke or TIA, were deemed as SBI.^24^

The locations of infarcts were further evaluated. For lacunar stroke, the presence of acute and silent infarcts in basal ganglia, corona radiata (anterior and posterior), thalamus, and infratentorial region were recorded respectively. For non-lacunar stroke, the presence of acute infarcts in the cortical (frontal, temporal, pariental and occipital lobes), corona radiata (anterior and posterior), basal ganglia, thalamus, and infratentorial region were recorded respectively. For all infarcts except infratentorial infarcts, the laterality was recorded as a left or right hemisphere. For patients with large infarcts covering more than one region, the presence of infarcts were deemed positive in all regions it covered.

### Assessment of PSD, function loss, and social support

Three months after the index stroke, another researcher blind to the clinical information administered the face-to-face interview. Patients were diagnosed as PSD if they presented symptoms described in the clinical criteria of the *Diagnostic and Statistical Manual of Mental Disorders*, 4^th^ edn (DSM-Ⅳ), and had a score ≥6 evaluated by the 15-item Geriatric Depression Scale (GDS) (scores ranging from 0 to15, a higher score suggesting a severer state of depression).^25^ Besides, the following information was collected: social support according to the Lubben Social Network Scale (LSNS) (scores ranging from 0 to 50, with a higher score indicating a lack of social support), which was designed for the evaluation of interactions between elderly and their social network, consisting of 10 items of different aspects of a social network; ^26^ functional status and degree of handicap according to the Modified Rankin Scale (mRS) (scores from 0 to 5, a higher score indicating unfavorable outcome).

### Statistical analysis

All data were analyzed with SPSS 21.0. As introduced above, patients were assigned to either the lacunar stroke group or non-lacunar stroke group. The comparison was performed between patients with and without PSD in each group respectively. Specifically, categorical variables were listed as proportions (numbers) and compared with the Chi-Square test between patients with and without PSD in lacunar and non-lacunar stroke groups respectively. Continuous variables were listed as mean ± standard error. The distribution of continuous variables was analyzed with the Shapiro-Wilk test. Student *t*-test and Mann-Whitney *U* test were used to compare the values of characteristics with or without normal distribution respectively between patients with and without PSD in each group. Variables with p<0.10 except GDS score were further added into multiple logistic regression models to identify the independent risk factors for the occurrence of PSD in each group; p<0.05 was considered to indicate the statistical difference.

## Results

Altogether 113 patients were lost to follow-up, and 431 patients were analyzed after follow-up, consisting of 246 patients with lacunar stroke and 185 patients of non-lacunar stroke. Compared with the 431 patients analyzed after follow-up, the 113 patients whose data were not analyzed after follow-up were older (p < 0.05) and had higher NIHSS scores (p < 0.05), with no significant difference in other characteristics.

### Lacunar stroke

83 (33.7%) patients of lacunar stroke were identified to have PSD. Compared with patients without PSD, patients with PSD were more likely to be female, with a higher prevalence of diabetes, severe neurological deficits, unfavorable treatment effects, a high degree of handicap, and low social support. The analysis of the locations of acute infarcts or all the infarcts showed no statistical difference between patients with and without PSD (Table 1).

**Table 1:** Characteristics of patients with lacunar stroke.

	**PSD (n = 83)**	**Non-PSD (n = 163)**	**p**
Age, years	69.53±7.68	69.21±7.84	0.798
Female	51 (61.4%)	74 (45.4%)	**0.017**
Education years	5.87±4.87	5.08±4.61	0.166
Hypertension	68 (81.9%)	123 (75.5%)	0.250
Diabetes	37 (44.6%)	50 (30.7%)	**0.031**
MMSE	25.11±2.17	25.72±2.81	**0.018**
NIHSS	4.63±1.89	3.51±1.76	**<0.001**
Unfavorable treatment effect	39 (47.0%)	39 (23.9%)	**<0.001**
mRS	2.60±1.45	1.73±1.20	**<0.001**
LSNS	33.53±4.65	35.45±5.22	**0.005**
GDS	8.81±2.12	2.19±1.34	**<0.001**
Grade of PWMH	1.28±1.02	1.12±1.01	0.247
Grade of DWMH	1.13±0.87	1.04±0.90	0.342
Presence of SBI	49 (59.0%)	88 (54.0%)	0.451
**Acute infarcts in**			
Left hemisphere	37 (44.6%)	70 (42.9%)	0.807
Right hemisphere	32 (38.6%)	73 (44.8%)	0.350
infratentorial	14 (16.9%)	20 (12.3%)	0.323
Basal ganglia	32 (38.6%)	62 (38.0%)	0.937
Anterior corona radiata	16 (19.3%)	38 (23.3%)	0.470
Posterior corona radiata	12 (14.5%)	32 (19.6%)	0.317
Thalamus	11 (13.3%)	25 (15.3%)	0.662
**Acute or silent infarcts in**			
Left hemisphere	57 (68.7%)	107 (65.6%)	0.634
Right hemisphere	50 (60.2%)	108 (66.3%)	0.352
infratentorial	31 (37.3%)	45 (27.6%)	0.118
Basal ganglia	45 (54.2%)	92 (56.4%)	0.740
Anterior corona radiata	27 (32.5%)	54 (33.1%)	0.925
Posterior corona radiata	28 (33.7%)	64 (39.3%)	0.397
Thalamus	29 (34.9%)	53 (32.5%)	0.703

A logistic regression model was constructed to identify the independent risk factors for the occurrence of PSD after lacunar stroke, with the items significant at the 0.10 level listed in Table 1 except GDS score added into the model. The results showed that females, a high NIHSS score, unfavorable treatment effect, high mRS, and low LSNS scores were independent risk factors for the occurrence of PSD (Table 2).

**Table 2:** Risk factors for the occurrence of PSD among patients with lacunar stroke.

	**OR (95% CI)**	**p**
Female	1.992 (1.074‒3.693)	**0.029**
Diabetes	1.536 (0.822‒2.872)	0.179
MMSE	0.892 (0.793‒1.004)	0.058
NIHSS	1.346 (1.125‒1.610)	**0.001**
Unfavorable treatment effect	3.260 (1.702‒6.245)	**<0.001**
mRS	1.410 (1.099‒1.810)	**0.007**
LSNS	0.934 (0.877‒0.995)	**0.035**

### Non-lacunar stroke

Among patients with non-lacunar stroke, 71 (38.38%) patients were identified to have PSD. Compared with patients without PSD, PSD patients were more likely to be female, with severe symptoms, a high degree of handicap. Besides, PSD patients had a higher prevalence of infarcts in the frontal and temporal cortexes (Table 3).

**Table 3:** Characteristics of patients with non-lacunar stroke.

	**PSD (n = 71)**	**Non-PSD (n = 114)**	**p**
Age, years	66.93±8.57	68.57±10.58	0.169
Female	42 (59.2%)	46 (40.4%)	**0.013**
Education years	5.96±4.00	5.83±4.29	0.669
Hypertension	51 (71.8%)	81 (71.1%)	0.909
Diabetes	29 (40.8%)	38 (33.3%)	0.301
MMSE	24.70±2.59	25.43±2.82	**0.034**
NIHSS	6.04±1.99	4.53±1.92	**<0.001**
Unfavorable treatment effect	30 (42.3%)	35 (30.7%)	0.109
mRS	3.06±1.22	2.32±0.93	**<0.001**
LSNS	32.38±4.70	33.08±4.17	0.343
GDS	8.49±1.56	2.48±1.47	**<0.001**
Grade of PWMH	0.96±0.92	1.04±1.04	0.710
Grade of DWMH	0.94±0.91	0.89±0.92	0.639
Presence of SBI	39 (54.9%)	58 (50.9%)	0.591
**Acute infarcts in**			
Left hemisphere	41 (57.7%)	55 (48.2%)	0.208
Right hemisphere	28 (39.4%)	51 (44.7%)	0.478
Infratentorial	9 (12.7%)	15 (13.2%)	0.924
Frontal cortex	29 (40.8%)	22 (19.3%)	**0.001**
Temporal cortex	30 (42.3%)	29 (25.4%)	**0.017**
Pariental cortex	15 (21.1%)	27 (23.7%)	0.686
Occipital cortex	8 (11.3%)	19 (16.7%)	0.312
Basal ganglia	17 (23.9%)	33 (28.9%)	0.456
Anterior corona radiata	16 (22.5%)	34 (29.8%)	0.278
Posterior corona radiata	20 (28.2%)	28 (24.6%)	0.586
Thalamus	3 (4.2%)	7 (6.1%)	0.575

A logistic regression model was constructed to identify the independent risk factors for the occurrence of PSD after non-lacunar stroke, with the items significant at the 0.10 level listed in Table 3 except the GDS score added into the model. The results showed that high NIHSS, high mRS scores, and the presence of acute infarctions in the frontal cortex were independent risk factors for PSD (Table 4).

**Table 4:** Risk factors for the occurrence of PSD among patients with non-lacunar stroke.

	**OR (95% CI)**	**p**
Female	1.956 (0.988‒3.875)	0.054
MMSE	0.900 (0.781‒1.038)	0.148
NIHSS	1.330 (1.082‒1.635)	**0.007**
mRS	1.514 (1.032‒2.221)	**0.034**
Acute infarctions in		
Frontal cortex	2.560 (1.109‒5.913)	**0.028**
Temporal cortex	1.733 (0.822‒3.653)	0.148

## Discussion

This study verified that depression was common after both lacunar stroke and non-lacunar stroke. The main determinants of PSD were different between lacunar and non-lacunar strokes to some extent. The severities of neurological deficits and handicaps were important for depression after both lacunar and non-lacunar infarction. Unfavorable treatment effects during hospitalization and lack of social support were critical for depression after lacunar stroke, while the lesion locations were more important for depression after non-lacunar stroke.

This study showed that the prevalence of PSD after lacunar stroke was around 1/3, close to that of PSD after non-lacunar stroke, and was similar with the results of previous reports about PSD.^27^, ^28^, ^29^ PSD after lacunar and non-lacunar stroke shared some common risk factors, such as female gender, degrees of neurological deficits and functional outcome, which had been studied and identified as the predictors of PSD for a series of studies.^29^, ^30^, ^31^, ^32^, ^33^, ^34^ Meanwhile, there were also some risk factors associated with PSD after lacunar stroke and non-lacunar stroke differently. For example, the unfavorable treatment effect was associated with PSD after lacunar stroke more strongly. Specifically, patients with lacunar infarctions who had no neurological functional improvement during hospitalization were much more likely to develop PSD than the rest (OR=3.260, p<0.001), and unfavorable treatment response or neurological deterioration were identified as the most important risk factor of PSD after lacunar stroke, while it wasn't that critical for PSD after non-lacunar stroke. There were no similar reports about the role of treatment effect or neurological deterioration in the mechanism of PSD. However, a recent study showed that the degree of disability at discharge was strongly associated with PSD.^29^ Compared with patients with non-lacunar stroke, patients with lacunar stroke usually have mild symptoms without severe physical disability at first, and therefore they might be physically and emotionally more sensitive to the deterioration of neurological function which could lead to relatively severe disability at discharge. However, this is just a hypothesis that requires more studies to prove it.

In this study, the authors made a detailed analysis of lesion location and found that the roles of lesion location in PSD were different between patients with lacunar and non-lacunar stroke. For patients with non-lacunar stroke, acute infarctions in frontal and temporal cortexes seemed to be associated with a high prevalence of PSD. However, the authors didn't get positive results about the laterality of infarcts which might be more likely to result in PSD. Furthermore, the analysis of lesion laterality and location among patients with lacunar stroke showed that neither the laterality nor the location of acute infarctions was associated with PSD. After the authors counted silent brain infarctions which were similar to symptomatic lacunar infarctions in many aspects,^24^ the results about the association between lesion location and PSD in lacunar infarction were still negative. Based on the results above, the authors concluded that frontal and temporal cortexes of both sides especially the former were critical locations for PSD. Actually, although the role of lesion location in the pathogenesis of PSD was still controversial, frontal lobe especially the left frontal lobe was mentioned most frequently in studies about the association between lesion location and PSD.^14^,^35^, ^36^, ^37^ Specifically, some studies suggested that the frontal cortex or the network of the limbic-cortical-striatal-pallidal-thalamic circuit which consists of both cortex and grey matters was crucial for the development of PSD.^12^,^36^,^38^ For lacunar stroke, the infarctions could be located in several places of the above-mentioned circuit including basal ganglia, thalamus, and anterior subcortex regions. However, the results of this study showed no significant association between lesion location and PSD after lacunar stroke. Based on this result, it seemed that the subcortical region might just contribute equally to the occurrence of PSD, unlike the frontal cortex which was proven to be associated with PSD more closely than other parts of the cortex.^12^,^36^ In the future, maybe functional MRI could supply more convincing details about the role of lesion location and PSD.

Silent cerebral lesions especially WML have been proven to be associated with the prevalence and severity of late-onset depression.^39^,^40^ According to the theory of “vascular depression”,^41^ the accumulation of silent lesions especially those in some critical regions might destruct the neurons and fibers involved in the process of mood regulation, thus leading to depression,^42^,^43^ similarly to the lesion location hypothesis of PSD. However, the present study showed that, in both lacunar and non-lacunar stroke groups, the degrees of PWML and DWML, and the prevalence of SBI had no statistical difference between patients with and without PSD. It suggested no significant association between specific locations of silent lesions and PSD. The authors speculate that the role of silent lesions on depression was overshadowed by the onset of stroke, i.e., the neurological deficits and the following handicap, which were more depressogenic. The similar deduction could also explain the different associations between social support and PSD in the two groups. Most previous studies proved the association between PSD and lack of social support.^44^,^45^ This study showed that this association mainly lay among patients with lacunar stroke. Compared with lacunar stroke, non-lacunar stroke usually results in severe neurological deficit which is a strong predictor of PSD and might weaken the influence of social support. This could be a possible explanation for the different associations mentioned above.

This was the first study about the significance of lesion location for the occurrence of depression after different subtypes of ischemic stroke. The main strength of this study mainly included the combined use of DSM-Ⅳ and GDS as the criteria of PSD, which could improve the specificity of diagnosis; and the comprehensive analysis of multiple factors including lesion location, silent lesions, stroke severity, treatment effect, functional outcome, and social support, which covered a large range of risk factors that might be associated with PSD. Meanwhile, there were also some limitations in this study. For example, the small sample size might limit the significance of the results. Besides, patients with symptoms too severe to give consent to this study or with severe aphasia (mostly non-lacunar stroke) were excluded from this study which lead to the imbalance of patient numbers in the two groups. Considering the strong association between stroke severity and PSD, the prevalence of PSD might be underestimated with some selective bias inevitable. In the future, more studies with a large sample size and elaborated design are still needed to explore the etiology of PSD.

## Statement of ethics

This study protocol was reviewed and approved by the Ethics Committee of Shanghai Xuhui Central Hospital, approval number (20190057). Written consent forms were obtained from participants or their family members.

## Authors’ contributions

Ke-Wu Wang: Was in charge of the data analysis and paper writing.

Yang-Miao Xu: Assisted with the data acquisition, data analysis and paper writing.

Chao-Bin Lou: Assisted with the data acquisition, data analysis and paper writing.

Jing Huang: Assisted with the data acquisition, data analysis and paper revision.

Chao Feng: Was in charge of the study design, data acquisition and paper revision.

## Funding sources

This paper was funded by grants from the Natural Science Foundation of Shanghai (No. 19ZR1450100) and the National Natural Science Foundation of China (No. 81971688).

## Declaration of Competing Interest

The authors declare no conflicts of interest.
